# Lipopolysaccharide responsiveness in vocal fold fibroblasts

**DOI:** 10.1186/s12950-014-0042-3

**Published:** 2014-12-19

**Authors:** Suzanne N King, Craig M Berchtold, Susan L Thibeault

**Affiliations:** Division of Otolaryngology–Head and Neck Surgery, Department of Surgery, University of Wisconsin-Madison, WIMR 5107 1111 Highland Avenue, Madison, WI 53705-2725 USA

**Keywords:** Vocal fold, Fibroblasts, Scar, Polyp, Toll-like receptors, Lipopolysaccharide

## Abstract

**Background:**

Vocal fold fibroblast’s (VFF) strategic location in the lamina propria and their ability to respond to external stimuli by producing inflammatory molecules suggest their possible direct involvement in innate immunity. Toll-like receptors (TLRs) are an essential signaling component to this response, as they allow for recognition of various microorganisms, leading to subsequent induction of pro-inflammatory genes. The objective of this study was to elucidate the role of VFF in the host immune response and subsequent influence on inflammatory cytokine secretion.

**Methods:**

VFF derived from polyp, scar, and normal tissue were treated with 5 μg/ml lipopolysaccharide (LPS). TLR1 through 9, CD14, and MD-2 were measured during stable conditions by polymerase chain reaction (PCR). Expression of TLR4 and IL-1R type-1 genes were quantified after 24 hrs LPS stimulation by reverse transcription-PCR. LPS responsiveness was determined by NF-κB nuclear translocation as measured by subunit p65 expression in nucleus with immunocytochemistry. Downstream effects were confirmed with immunoassay measuring IL-8 concentrations in supernatant after 8 hrs.

**Results:**

All VFFs constitutively expressed TLR1 to 6, TLR9, CD14, and MD-2 mRNA. Polyp VFF exhibited significantly higher TLR4 transcript levels (p < 0.001) in comparison to scar and normal VFF. LPS stimulated scar and polyp VFF exhibited increased levels of p65 in the nucleus (p < 0.01) and secreted greater IL-8 protein (p < 0.0001) compared to normal VFF.

**Conclusion:**

VFF constitutively express genes for the receptors essential to the host immune response. Scar and polyp VFF produced greater LPS responsiveness resulting in over-activated inflammatory patterns. These findings support VFF role in the pathogenesis of inflammatory vocal fold disorders and suggests their presence in the wound bed could lead to chronic inflammation.

## Background

Chronic trauma to the vocal folds from vocal use, reflux, bacteria, or viral pathogens can influence inflammation and tissue repair, contributing to the pathophysiology of benign vocal fold lesions [1-3]. Recent evidence has suggested that exposure to these types of challenged microenvironments can cause vocal fold fibroblasts (VFF) to undergo behavior changes that can alter their inflammatory phenotype [4]. In the vocal folds, fibroblasts are highly abundant and strategically located in the most superficial layer of the lamina propria [5] where infection or physical damage occurs. Although VFF are known to be involved in tissue homeostasis [6,7] and immune cell regulation [8] their ability to directly recognize immune challenges remains unclear. Whether this response differs between normal cells and those taken from inflammatory or scarred vocal fold lesions also warrants investigation, as a possible diagnostic or therapeutic target.

For fibroblasts to directly contribute to the innate immune response they require pattern-recognition receptors (PRR) such as Toll-like receptors (TLR), which bind to the repetitive structural components on the outer layer of microorganisms. PRR provide an element of specificity to innate immune cells as they help distinguish between self and non-self, which would otherwise require gene rearrangement and clonal expansion (i.e. adaptive immunity). TLRs are an essential signaling component of the innate immune response, because ligand binding activates the transcriptional factor nuclear factor (NF)-ĸB leading to the induction of pro-inflammatory cytokine genes (i.e. IL-8) that can regulate the response to infection [9]. The NF-κB protein consists of a heterodimer (p65/p50), which is constitutively localized to the cytoplasm by its inhibitor, IκBα while the inflammatory stimulus induced degradation of IκBα allows NF-κB to translocate to the nucleus for the de novo synthesis of genes regulating inflammation. To date, ten members of the human TLR family have been identified (labeled 1–10) with nine of these receptors having known ligands such as, bacteria (TLR2, 4 & 6), fungi (TLR5 & 6), and viruses (TLR7 & 8).TLRs are transmembrane proteins with leucine rich repeat domains in the extracellular region and intracellular Toll/IL-1 receptor (TIR) domain in the cell’s intracellular compartment. TLR4 is the most researched and several different stromal and immune cells express its functional form, including fibroblasts and epithelial cells [10,11]. It can play a critical role during host defense, because of its recognition of gram-negative bacteria and endogenous ligands expressed after tissue injury. Additionally, recent evidence has shown that TLR4 gene and protein levels are upregulated in fibroblasts taken from hypertrophic scars [12].

There is increasing clinical evidence to support the link between bacterial flora and benign vocal fold lesions, such as polyps [2]. For instance, helicobacter pylori a gram-negative bacteria is most commonly found in the stomach, but evidence has shown its laryngeal presence in patients diagnosed with vocal fold polyps [1]. Host responsiveness to this unusual microbe in the larynx is unclear. TLR4 and its accessory molecules CD14 and MD2 recognize and respond to the cell wall of gram-negative bacteria, which is composed of a distinct lipopolysaccharide (LPS) chain [13]. Recent work by our group has analyzed VFF influence on the function of surrounding immune cells after exposure to LPS [8]. We demonstrated that VFF derived from polyp, scar, and normal tissue can modulate macrophages paracrine signaling during early cytokine expression (i.e. TNF-α, IL-10, IL-12) and subsequent chemokine and growth factor expression (i.e. IL-6, IL-8, MCP-1, TGF-β). This work suggests that VFF are a component of the innate immune response capable of determining the quantity and duration of macrophages response [8]. However, VFF direct contribution in regulating host defense remains undefined.

The objective of the present *in vitro* study was to elucidate the role of VFF in the host immune response and subsequent influence on inflammatory cytokine secretion. First, we hypothesized that VFF constitutively express TLRs and TLR4 accessory genes, which are required for direct recognition of various viral and bacterial pathogens and foreign antigens. Second, we hypothesized that VFF derived from polyp and scar tissue produce functional TLR4 after ligand stimulation resulting in high cytokine secretion downstream where as normal VFF are minimally responsive to stimuli.

## Methods

### Vocal fold fibroblasts

Human fibroblasts cell lines were derived from polyp, scar, and two age-matched normal vocal fold tissue samples based on protocols approved by the University of Wisconsin Health Sciences Institutional Review Board as previously described [8,14,15]. Cells were cultured in Dulbecco’s Modified Eagle Medium (DMEM) supplemented with 10% fetal bovine serum (FBS), 100 U/mL penicillin, 0.01 mg/mL streptomycin sulfate and 1× non-essential amino acid (all from Sigma Inc, St Louis) at 37°C in atmosphere of 5% CO_2_. Fibroblasts were used between passages five and nine.

### RNA isolation

Normal (T21 and T59), polyp, and scar VFF were seeded in triplicate on 6-well plates at 3 × 10^5^ per well (Becton Dickinson Labware) and cultured until 80% confluent. Cells were then treated with or without 5 μg/mL LPS (γ-irradiated, escherichia coli O26:B6, Sigma-Aldrich, USA) for 24 hrs. Total cellular RNA was extracted from VFF using Rneasy Mini Kit (Qiagen, Valencia, CA) according to the manufacturer’s instructions and as previously described [16]. First strand cDNA was synthesized from 1 μg of total RNA using an Omniscript Reverse Transcription Kit (Qiagen) and random primers (Integrated DNA Technologies, Coralville, IA).

### Polymerase chain reaction for transcription of TLRs and TLR4 accessory molecules

To determine if VFF express genes necessary for pathogen recognition, we measured the gene expression of nine TLRs and TLR4 accessory molecules CD14 and MD-2 during steady state conditions. GoTaq Hot Start Polymerase (Promega Corporation, Madison, WI) was used with the PTC- 200 Peltier Thermal Cycler according to the manufacturer’s protocol. Primer sequences, gene bank access numbers, and expected PCR product sizes are listed in Table 1. Specificity of each primer pair was confirmed by showing a single peak and appropriate sized DNA band for each gene product. Amplifications were optimized for each primer and carried out for 40 cycles: denaturing at 95°C for 30 seconds, primer specific annealing temperature for 45 seconds (Table 1), and extension at 72°C for 2 minutes. Reaction yields were determined by electrophoresis on 2% agarose gel using 12 μl of each PCR product and visualized by ethidium bromide staining. U937 (ATCC, VA; CRL1593.2) cells treated with or without tumor necrosis factor (TNF)-α or LPS were positive controls. H2O was used in place of template cDNA as a negative control and included in each experiment.

**Table 1:** **Primer Sequence and Products of Polymerase chain reaction (PCR)**

**Name**	**GenBank #**	**Forward**	**Reverse**	**Size**
TLR- 1	NM_003263	CTATACACCAAGTTGTCAGC	GTCTCCAACTCAGTAAGGTG	220
TLR- 2	NM_003264	ACCTGTCCAACAACAGGATCACCT	TGTTCAAGACTGCCCAGGGAAGAA	139
TLR- 3	NM_003265	ACTGAACCATGCACTCTGTTTGCG	TGACGAAAGGCACCTATCCGTTCT	96
TLR- 4	NM_138554	ATGCAGGGCTGCTAATCTCAAGGA	GTTGGTTGAAATGCCCACCTGGAA	93
TLR- 5	NM_003268	GTTGCAACTTGCCTGGGAAACTGA	TCAGCCTGTTGGAGTTGAGGCTTA	164
TLR- 6	NM_006068	AGGTGCCTCCATTATCCTCA	GAATCCATTTGGGAAAGCAG	211
TLR- 7	NM_016562	AATGTCACAGCCGTCCCTACTGTT	TTTACACGGCGCACAAGGAAATGG	180
TLR- 8	NM_138636	TGTCTCAGAGGCTGCAATGTAGGT	AGGCTCGCATGGCTTACATGAGTA	136
TLR- 9	NM_017442	ATCTGCACTTCTTCCAAGGCCTGA	AGAAGGCCAGGTAATTGTCACGGA	144
IL-1R, type 1	NM_000877.2	AGAGGAAAACAAACCCACAAGG	CTGGCCGGTGACATTACAGAT	106
MD-2	NM_015364.4	TTGCCGAGGATCTGATGA	GGTGTAGGATGACAAACTCC	185
CD14	NM_001174105	TGCCGCTGTGTAGGAAAGAAGCTA	AGACGCAGCGGAAATCTTCATCGT	182

### Real-time polymerase chain reaction for TLR4 responsiveness

To compare polyp, scar, and normal (T21 & T59) VFF production of TLR4 after direct ligand stimulation, we treated cells with or without LPS. IL-1 receptor type I (IL-1R1) mRNA was also measured to determine its effects with LPS stimulation because autocrine IL-1β signaling can induce NF-ĸB and enhance IL-8 secretion [17]. FastStart DNA Master SYBR Green I kit (Roche) with the LightCycler System was used to quantify samples as previously described [6,16]. Amplification of β-actin was used as an internal control. Amplification efficiencies of target and reference genes were assessed by LightCycler software and specificity of each pair of primers was confirmed by melting curves. A diluted PCR product was used to assess the PCR replication efficiency for all genes. U937 cells were used as positive controls as described above at the same concentration of total RNA as VFF. Standard curves were derived to quantify gene expression and concentrations were normalized to β-actin housekeeping gene.

### Immunocytochemistry for NFκB translocation

To determine the functionality of TLR4 in VFF, we measured the nuclear translocation of RelA (NF-ĸB subunit p65) after LPS induction in accord with previous studies [18]. Rel A (p65) contains a transcriptional activation domain in the carboxyl terminus of the protein necessary for gene activation [19]. Normal (T21 and T59), polyp, and scar VFF were seeded at 1.5 × 10^5^ per well into 12 well plates (Becton Dickinson Labware) containing sterile glass cover slips (Fisherbrand, 15CIR-1D; Fisher Scientific, Pittsburg, PA). Cover slips were incubated with DMEM-10% FBS twenty-four hours prior to improve cell adherence. U937 cells stimulated with 32 nM TPA (phorbol, 12-myristate, 13 Acetate) for 48 hrs to induce monocyte/macrophage differentiation (cell adhesion) were used as controls with and without LPS stimulation. After six hours of incubation, fibroblasts were serum starved for twenty-four hours to synchronize the cell cycle at G_0_ [20]. Cells were then stimulated by adding DMEM-10% FBS supplemented with 5 μg/ml LPS for 30 and 45 minutes. These time points were chosen to ascertain the appropriate activation kinetics with LPS [21]. Cover slips were then washed with phosphate buffered saline (PBS; Sigma-Aldrich) and fixed with 4.0% formaldehyde (Polysciences, Inc., Warrington, PA) for 30 minutes. Cells were then washed with wash buffer (PBS- 0.1% Triton X-100 [Sigma-Aldrich]) and permeabilized with 0.5% Triton X-100 in PBS. After 5 minutes, cells were incubated with blocking buffer (PBS, 1% bovine serum albumin [BSA], 1% gelatin, 10% goat serum; all from Sigma-Aldrich) for 30 min. Cells were then incubated with anti-p65 rabbit polyclonal IgG diluted 1:100 for 60 min (Santa Cruz Biotechnology, USA) followed by incubation with fluorescein isothiocyanate (FITC) conjugated goat anti-rabbit secondary antibody diluted at 1:200 (Invitrogen, Life Technologies, Grand Island, NY). After 60 minutes, cover slips were washed and mounted to glass slides (Premium, Fisher Scientific) with Vector aqueous anti-fade fluorescent medium with 4′,6-diamidino-2-phenylindole (DAPI; Vectashield; Vector Laboratories, Burlingame, CA). All procedures were performed at room temperature. Secondary antibody alone was run as a negative control.

Images were captured using a Nikon Eclipse E600 Epi Flourescence inverted microscope (Nikon, Tokyo, Japan) at 200× magnification and subsequently analyzed for the presence of NF-ĸB (green) and nuclear staining (blue) at the same exposure time (green 1/1.2 seconds, blue 1/40 excitation). To avoid biases with NF-κB staining, images were taken by person unfamiliar to the study and selected for analysis by viewing nuclear staining first to identify regions of confluent cells. Data acquisition was carried out with ImageJ software. Three images were taken per slide. Total cell counts were determined for each nuclear stain (DAPI) image by producing binary versions of the image and using plugins to analyze and automatically count each particle. Manual cell counts were taken with the Fiji Crosshair plugin from unidentified images to determine NF-κB translocation. Cells were considered NF-κB positive if bright green fluorescence was localized in the nucleus. Percentages of positive cells per image were determined by dividing the number of NF-κB positive cells to the total cell count of the image.

### Enzyme-linked immunosorbent assay (ELISA) for Cytokine Secretion

To determine the direct downstream signaling effect after TLR4 activation in VFF, we measured the production of IL-8 pro-inflammatory cytokine after LPS stimulation. VFF were seeded at near confluence 2 × 10^5^ per well into 12 well plates (Becton Dickinson Labware) to control for growth kinetics. Following 24 hrs of serum starvation, DMEM-10% FBS supplemented with or without 5 μg/mL LPS was added to each well. After 8 hrs incubation, supernatants were removed and stored at −80°C. Protein secretion levels were measured using an IL-8 ELISA according to the manufacturer’s protocol (Invitrogen, USA). Standard curves were used to measure the quantity of each sample. Non-stimulated cells and U937cells stimulated with and without LPS were used as controls.

### Statistical analysis

All experiments were performed in triplicate and data expresses the mean ± standard deviation (SD). One-way analysis of variance (ANOVA) was used to compare differences in gene expression levels between cell type and treatment group. A repeated measure ANOVA was used to analyze differences in NF-κB expression between group, day, and group by day interaction. If the F-test revealed significant differences at the 0.05 level, pairwise comparisons were used to determine statistical differences between samples (Fisher’s least squares means). A Pearson correlation coefficient was used to analyze the relationship between the mean of IL-8 expression and the two NF-κB time points. A p-value of <0.05 was considered significant.

## Results

### Differences between VFF constitutive expression of TLR 1–9

**Figure 1:**
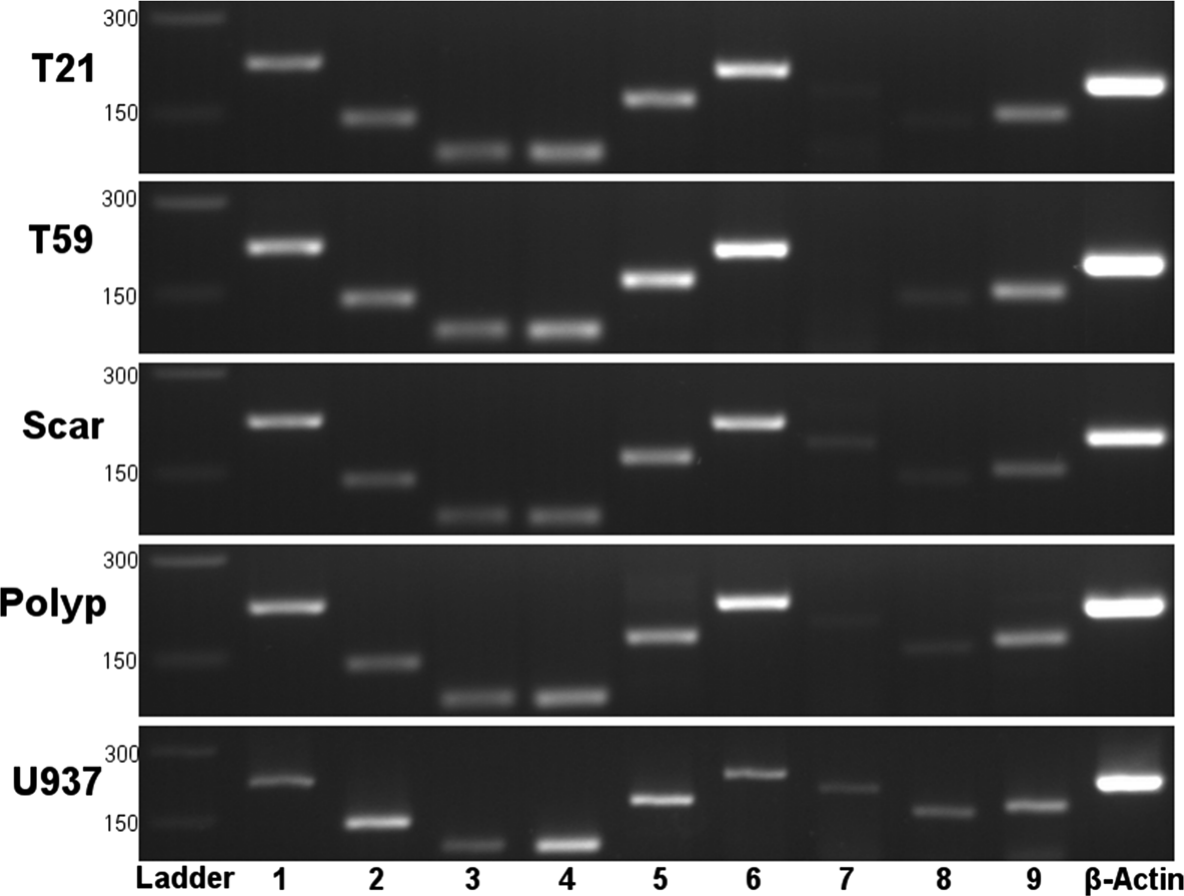
**Constitutive mRNA expression of TLR 1 to 9 in VFF.** Polymerase chain reactions showed that scar, polyp, and normal (T21 and T59) VFF expressed TLR 1 to 6, TLR8, and TLR9 genes during steady state conditions. Amplification of β-actin was used as an internal control. Appropriate sized DNA band for each gene product was determined by 100 bp ladder. Panels depict individual VFF and the PCR product of each TLR is displayed in lanes marked 1 through 9.

**Figure 2:**
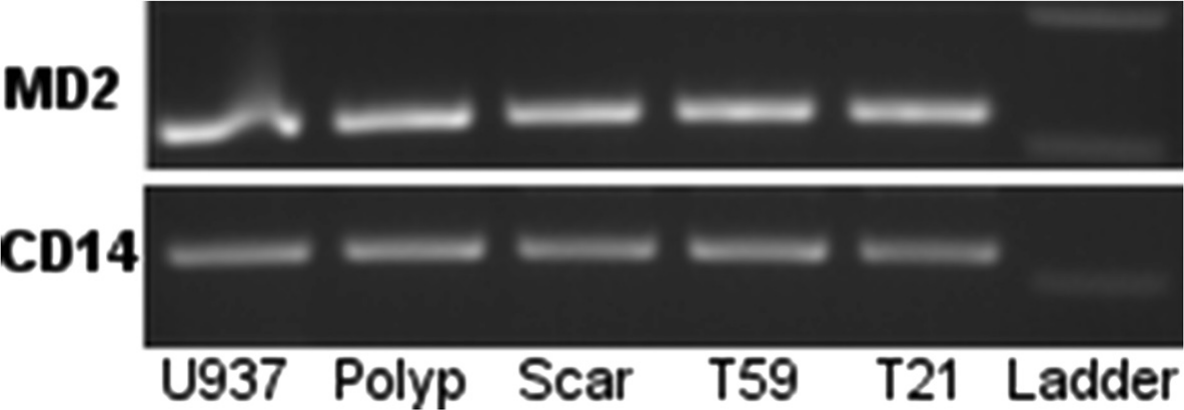
**Constitutive mRNA expression of TLR4 accessory molecules in VFF.** Polymerase chain reactions showed that scar, polyp, and normal (T21 and T59) VFF expressed MD-2 and CD14 mRNA. Appropriate sized DNA band for each gene product was determined by 100 bp ladder. U937 cells were used as a positive control.

To determine patterns of TLR gene production during steady state conditions and to confirm TLR4 accessory molecule expression, cDNA from scar, polyp, T21, and T59 VFF were amplified for target genes and analyzed using gel electrophoresis. VFF demonstrated expression of TLR1 through TLR6 and TLR9 with noticeable increases in the product size of TLR5 and TLR6 in comparison to all other receptors (Figure 1). No noticeable product is present for TLR7 in normal VFF samples, however a faint expression is appreciated with scar and polyp VFF. TLR8 is also absent in T21 normal VFF and only a faint expression noted in T59, scar, and polyp VFF. All VFF expressed similar MD2 and CD14 transcription levels (Figure 2).

### Differences in VFF gene expression after stimulation with TLR4 ligand

**Figure 3:**
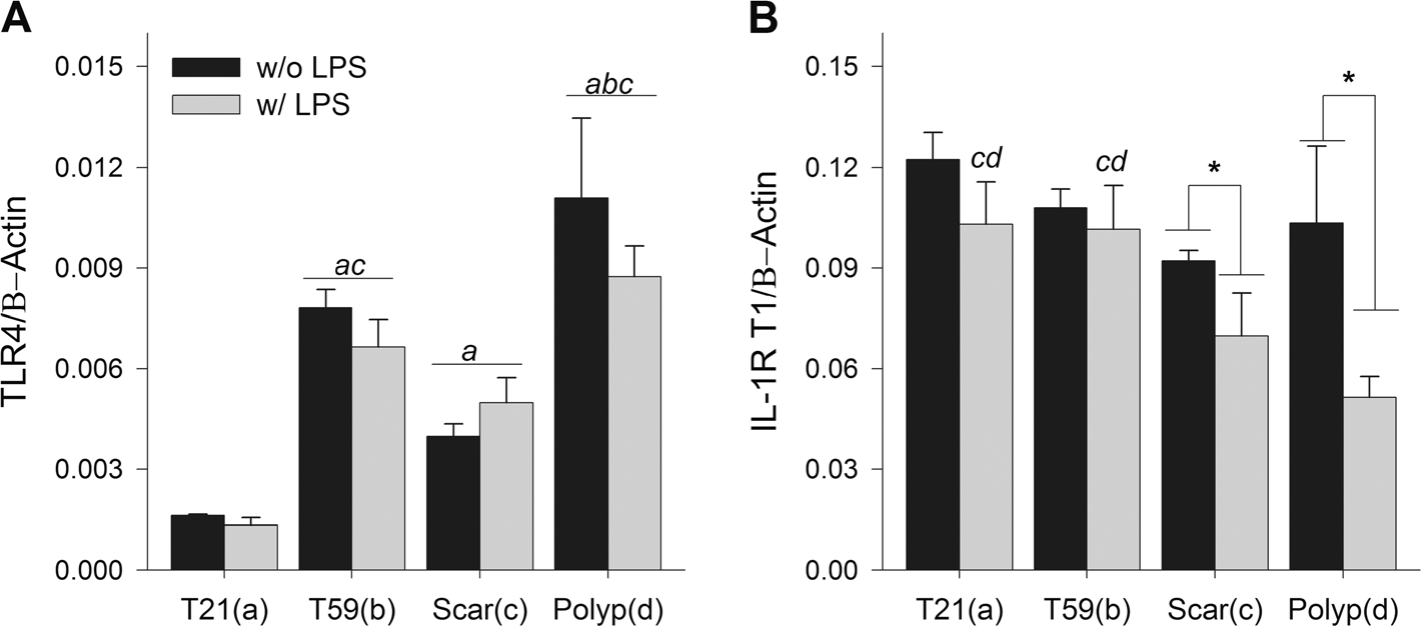
**Comparison of normal, scar, and polyp VFF gene expression after LPS stimulation.** Cells were treated with 5 μg/mL LPS for 24 hrs. RNA was subsequently extracted, converted into cDNA, and then analyzed for **(A)** TLR4 and **(B)** IL-1 receptor 1 gene expression using RT-PCR. Amplification efficiencies of target and reference genes were assessed by LightCycler software. Standard curves were derived to quantify gene expression and concentrations were normalized to β-actin housekeeping gene. Letters represent a statistical significance of p < 0.05 when compared to (a) T21 (b) T59 (c) scar (d) polyp. *represents differences within cell type with (w/) LPS compared to without (w/o) LPS.

To characterize if TLR4 and IL-1R1 gene expression is susceptible to LPS stimuli, we treated cells for 24 hrs and analyzed them with RT-PCR. Concentrations for each gene were determined by relative standard curves. Results were normalized to the housekeeping gene by calculating the ratio of the target gene to the housekeeping gene. Data is presented in Figure 3 as fold-change. Stimulation with LPS did not affect VFF constitutive expression of TLR4 gene. However, differences in TLR4 expression were found across types of VFF. Polyp VFFs were found to have statistically higher TLR4 gene in comparison to all other VFF (T21 and scar p < 0.0001; T59 p < 0.001). T59 VFF also demonstrated statistically higher expression in comparison to T21 (p < 0.0001) and scar VFF (p < 0.001). LPS induced TLR4 ligation altered VFFs expression of IL-1R1 gene, which is an important regulatory mechanism of the inflammatory response. Normal T21 and T59 VFF expressed significantly higher levels of IL-1R1 after LPS stimulation in comparison to similar conditions with scar (p < 0.01) and polyp VFF (p < 0.0001). Significant decreases in IL-1R1 gene were also found when polyp (p < 0.0001) and scar (p < 0.05) were treated with LPS in comparison to controls.

### LPS stimulated sustained upregulation of NF-ĸB in Polyp and Scar VFF

**Figure 4:**
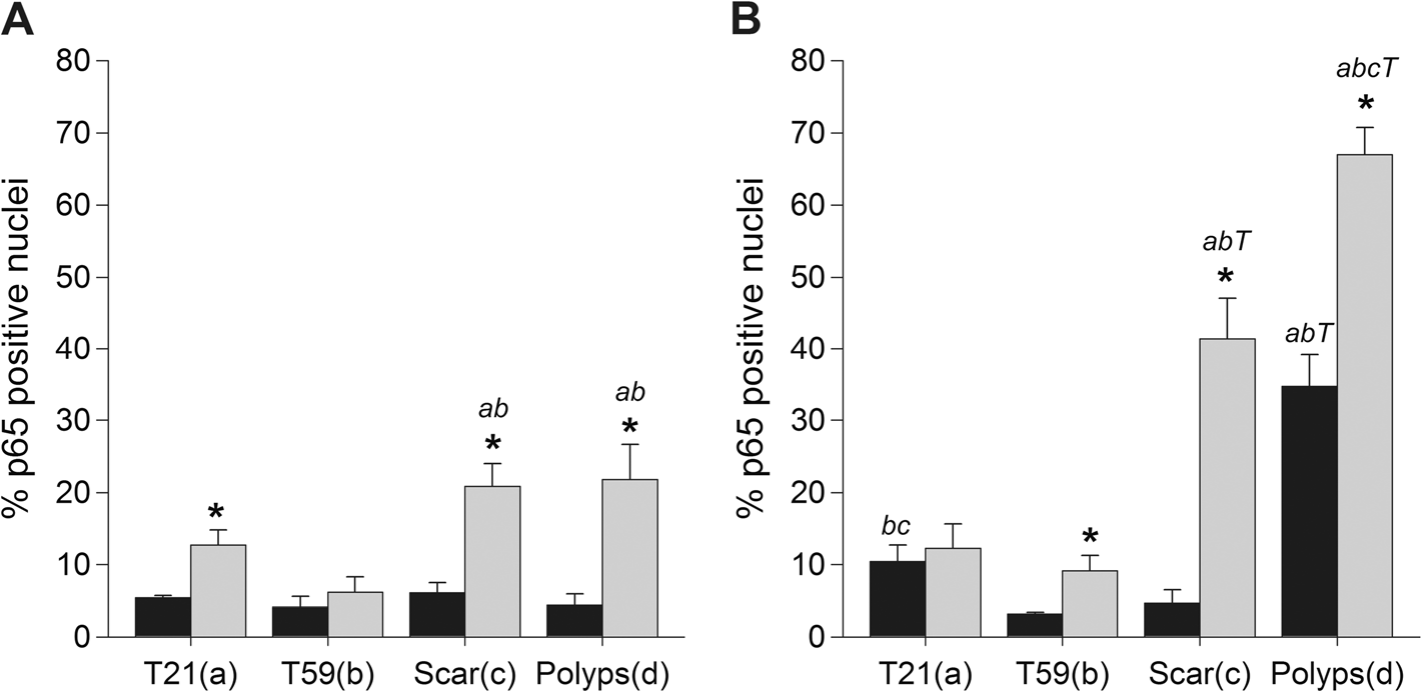
**Vocal fold fibroblasts NF-κB translocation into the nucleus.** Normal, scar, and polyp VFF were seeded on cover slips at 1.5 × 10^5^ cells and stimulated with or without 5 μg/ml of LPS for 30 min **(A)** or 45 min **(B)**. Cells were stained for NF-κB p65 translocation into the nucleus and nucleic acids. Data is graphed as the mean of the expression % of total cells counted ± SD. Letters represent a statistical significance of p < 0.05 when comparing (a) T21 (b) T59 (c) scar (d) polyp across similar conditions. T represents differences between 30 and 45 min time points compared within treatment groups. *p < 0.05 represent differences within cell type with (w/) LPS compared to without (w/o) LPS within each cell type.

**Figure 5:**
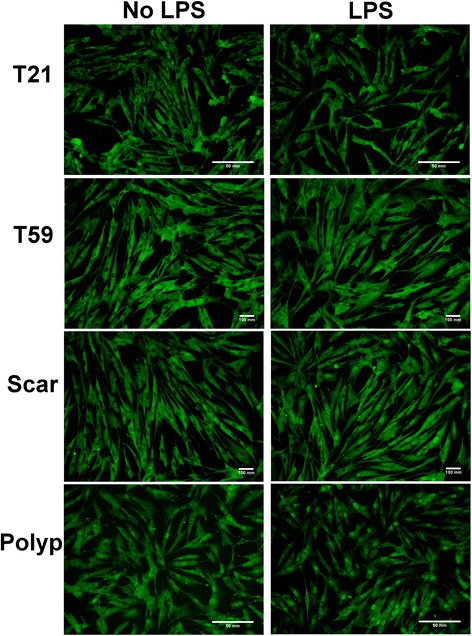
**LPS stimulated upregulation of NF-ĸB in VFF** ***.*** Scar and polyp vocal fold fibroblasts (VFF) stimulated with lipopolysaccharide (LPS) for 45 minutes had higher percentage of NF-κB activation as detected by immunocytochemistry. NF-κB p65 translocation into the nucleus was determined by immunofluorescence. The first column denotes normal (T21 & T59), scar, and polyp VFF baseline percentage levels without LPS and second column shows levels after LPS stimulation.

To confirm the functional activation of TLR4 ligation, NF-ĸB nuclear translocation was measured by assessing p65 protein expression in the nucleus after 30 and 45 minutes of LPS stimulation. Results are presented in Figures 4 and 5. T21 (p = 0.01), scar (p < 0.0001), and polyp VFF (p < 0.0001) had a statistically higher percentage of p65 positive nuclei after 30 minutes of LPS-induction compared to non-stimulated controls whereas no differences were found within T59 VFF conditions. Nuclei with p65 staining were significantly greater in T59 normal VFF (p < 0.02), scar (p < 0.01), and polyp (p < 0.01) conditions at 45 min post-LPS compared to their non-stimulated controls. No significant differences were found 45 minutes post LPS with T21 VFF. Temporal differences were observed with polyp and scar VFF, demonstrating increased percentage of p65 positive nuclei (p < 0.0001) after 45 minutes of LPS stimulation in comparison to the 30 minute time point. No temporal differences were seen with normal VFF.

### Influence of TLR4 ligation on cytokine expression

**Figure 6:**
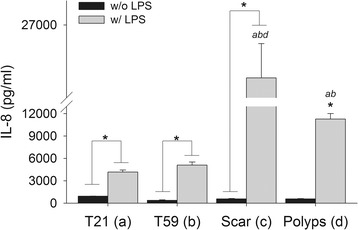
**Downstream cytokine effects of TLR4 ligand activation.** Vocal fold fibroblasts (VFF) exhibited increased expression of IL-8 after TLR4 activation. Polyp, scar, T21 and T59 VFF were seeded in 12-well plates at 2 × 10^5^ cells/well. After 8 hrs of stimulation with 5 μg/ml of LPS, cell culture supernatants were analyzed for IL-8 expression levels. Data is graphed as the mean concentration (pg/ml) ± SD. Numbers represent a statistical significance of p < 0.001 when compared to (a) T21 (b) T59 (c) scar (d) polyp. *p < 0.05 represent differences within cell type with (w/) LPS compared to without (w/o) LPS within each cell type.

To determine the downstream effects of TLR4 ligand activation, the secretion of IL-8 an acute pro-inflammatory cytokine well established to be induced by NF-κB activation with LPS in fibroblasts was measured after LPS stimulation for 8 hours [22,23]. Results presented in Figure 6. After LPS induction, VFF derived from polyp, scar, and normal (T21 and T59) tissue demonstrated a significant upregulation in IL-8 expression (p < 0.0001) in comparison to untreated controls. Across LPS treated conditions, scar VFF demonstrated statistically higher expression of IL-8 in comparison to polyp, T21, and T59 VFF (p < 0.0001). Polyp VFF exhibited significantly higher levels of IL-8 expression after LPS stimulation in comparison to similar conditions with T21 and T59 VFF (p < 0.0001). T59 VFF also secreted significantly more IL-8 in comparison to T21 VFF (p < 0.02).

### Relationship between NF-ĸB activation and downstream cytokine secretion levels

To characterize the correlation of VFF activation after LPS stimulation, we compared the mean percentage of p65 positive nuclei with mean IL-8 secretion levels. The percentage of positive nuclei with VFF (polyp, scar, and normal) after 30 minutes of LPS activation significantly correlated to IL-8 secretion (0.844, p < 0.01). No significant associations were noted at the 45 minute time point (0.601, p = 0.115).

## Discussion

The larynx is located at the crossing of the respiratory and gastrointestinal systems exposing the vocal folds to a diverse repertoire of microorganisms. During decline of epithelial barrier function [24-26] (e.g. reflux, dehydration, phonotrauma), microflora on the luminal surface of the vocal fold may permeate the epithelium and activate an innate immune response in the lamina propria. TLRs are critical components to this response, quickly detecting the conserved molecular patterns on microbial surfaces (PAMPs), which signals an antimicrobial defense (i.e. cytokines, phagocytes, complement cascade) and controls the initiation of the adaptive immune response. Recent evidence has demonstrated that fibroblasts from other areas of the body can directly contribute to innate immune response by producing functional TLRs [11,12,27]. Since fibroblasts are the most prominent cells in the vocal fold lamina propria and likely one of the first to encounter infection, understanding their innate immune recognition patterns and their function in response to a known immunologic adjuvant is essential. Overall, our results are novel as they demonstrate that human VFF from normal and pathologic tissue present marked differences in response to inflammatory stimuli.

Several cell types found in soft tissue express TLRs genes and their accessory molecules, including dermal fibroblasts, endothelial, keratinocytes, epithelial, monocytes, macrophages, and dentritic cells [10,12,27,28]. However, because regions of the body with higher exposure to external environment (i.e. lung, gastrointestinal tract) have been shown to exhibit increased TLR mRNA [29,30], patterns of TLR expression may differ across cell source. The continuous exposure to pathogens and commensal microorganisms in the upper respiratory tract, which includes the vocal folds, would suggest the need for TLR signaling to maintain homeostasis. In the present study, we found that all VFF constitutively expressed TLR1 to 6, TLR9, and accessory molecules CD14 and MD2 genes, which are needed to directly recognize bacteria, fungi, and viruses. To our knowledge this is the first report of TLR and accessary molecule expression specific to the vocal folds. Further investigation is necessary to determine their potential role in regulating normal vocal fold homeostasis.

Given that exogenous and endogenous ligands are capable of activating TLRs, tight regulation of these signal pathways is vital. Our findings demonstrated that TLR4 gene expression was highest in fibroblasts derived from polyps irrespective of LPS stimulation. It has been hypothesized that vocal fold polyps are caused by the inflammatory response to vascular injury [31] and are sometimes exposed to various microorganism, such as gram-negative bacteria [1]. Under these pathologic conditions, surrounding cells can undergo damage or stress, as the environment is enriched with inflammatory mediators (i.e. COX-2, HIF-1α) [32,33], proteases (MMP-2 ,-9) [33], and ECM proteins (i.e. fibronectin, decorin) [31,34,35] that can produce endogenous ligands and subsequently activate TLRs (i.e. TLR2 or TLR4). Thereby, altering downstream signaling and influencing the inhibitory mechanisms that are in place to protect against persist inflammation [12]. TLR4 profiles could be used to distinguish vocal fold disorders with similar macroscopic appearances (i.e. reinke’s edema, polyp, cyst), which are known to be difficult to clinically ascertain [36]. However, a limitation of our study is the lack of biologic replicates of human vocal fold samples. Therefore, confirmation of these findings is necessary both amongst similar inflammatory vocal fold lesions and *in vivo* with multiple donors.

Resolution of inflammation requires rapid induction and resolution of NF-ĸB after LPS signaling, to control the transcription of inflammatory genes needed to fight a bacterial infection. Innate immune cells (i.e. macrophages) exhibit peak NF-ĸB activation after thirty minutes that quickly drops off during subsequent time points [22]. In contrast, fibroblasts have been found to exhibit delays in NF-ĸB activation leading to peaks at later time points [21]. This is likely associated to the need for additional adaptor molecules i.e. MyD88 and TIR-containing adaptor molecules (Mal/TIRAP) during signaling [22]. In our study, scar and polyp VFF physiologic response to LPS resulted in a higher percentage of NF-ĸB activated cells in comparison to the minimal responses found with normal VFF. Furthermore, when polyp and scar VFF were stimulated with LPS they exhibited a down regulation of IL-1R1 mRNA, which may suggest that the observed effects of NF-ĸB and IL-8 was produced in response to TLR4 ligation as opposed to autocrine signaling stimulation by IL-1β. These findings demonstrate that VFF from pathologic tissue respond more robustly to LPS stimulation as indicated by persistent increases in NF-ĸB activation and IL-8 secretion. It is known that fibroblasts taken from pathologies exhibit unique phenotypic characteristics [37] and therefore, differences found may show the potential education of fibroblasts in polyp and scar vocal folds. From our work it is unclear if the responsiveness to LPS is due directly to NF-ĸB activity. NF-ĸB overactivation has been implicated in several inflammatory diseases, including rheumatoid arthritis or atherosclerosis [38]. Targeting NF-ĸB activity with steroids is a commonly used clinical approach in Otolaryngology Head and Neck Surgery to treat inflammatory disorders, but there is disagreement in its effectiveness [39-42]. Our results would suggest that glucocorticoids may only be effective in the presence of inflammatory stimuli in the vocal fold since there is no evidence for constitutive activation of NF-ĸB in the four donor cell lines studied. However, given that steroids can adversely affect wound healing, treatments with steroids may potentially delay the inflammatory response against the insulting agent (i.e. LPS). Further investigation into the effectiveness of steroids in down regulating VFF inflammatory responsiveness is necessary and warranted before clinical recommendations could be made.

Impaired TLR4 expression can alter the signaling cascade, leading to a persistent pathologic state. Downstream effects of LPS signaling resulted in elevated IL-8 expression in polyp and scar VFF, which strongly correlated with the percentage of NF-ĸB activated cells after 30 minutes. Scar VFF exhibited the highest IL-8 cytokine expression overall, which warrants further discussion. One would speculate that fibroblasts derived from vocal fold polyps would be more likely to produce higher amounts of IL-8 due to the inflammatory nature of the pathology [32,33], as well as the higher constitutive levels of TLR4 gene and greater percentage of NF-ĸB activated cells that we observed in the current study. These downstream discrepancies suggest different regulatory proteins may be controlling the expression in each cell type, possible mediating the disorder. We previously reported that co-culturing polyp VFF with activated macrophages resulted in significantly more IL-8 secretion than similar co-cultures conditions with scar VFF [8]. These studies lend further support to the notion that fibroblasts isolated from scar or polyp lesions undergo pathology-induced education.

## Conclusion

The results of this study demonstrate that human VFF from pathologic tissue present marked differences in response to inflammatory stimuli. This work outlines a rational paradigm for clinical assessment of the TLR4 pathway not only to improve clinical diagnosis, but also to expand treatment options by analyzing its effects on the resolution of inflammation.

## Abbreviations

VFF: Vocal fold fibroblast’s
TLRs: Toll-like receptors
LPS: Lipopolysaccharide
PCR: Polymerase chain reaction
IL-8: Interleukin
PRR: Pattern-recognition receptors
NF-ĸB: Nuclear factor
TIR: Toll/IL-1 receptor
TNF-α: Tumor necrosis factor
MCP-1: Monocyte cheomattractant protein
TGF-β: Transforming growth factor
DMEM: Dulbecco’s Modified Eagle Medium
FBS: Fetal bovine serum
IL-1RI: IL-1 receptor type I
PBS: Phosphate buffered saline
DAPI: 4′,6-diamidino-2-phenylindole
ELISA: Enzyme-linked immunosorbent assay
SD: Standard deviation
PAMP: Patterns on microbial surfaces
COX-2: Cyclooxygenase
HIF-1α: Hypoxia-inducible factor
MMP-2: Matrix metalloproteinase
ECM: Extracellular matrix
